# Inkjet-printed flexible planar Zn-MnO_2_ battery on paper substrate

**DOI:** 10.1038/s41598-024-51871-5

**Published:** 2024-01-18

**Authors:** Sagnik Sarma Choudhury, Nitish Katiyar, Ranamay Saha, Shantanu Bhattacharya

**Affiliations:** 1 Microsystems Fabrication Laboratory, Indian Institute of Technology, Kanpur, 208016 India; 2 Department of Mechanical Engineering, Indian Institute of Technology, Kanpur, 208016 India

**Keywords:** Energy science and technology, Engineering, Materials science

## Abstract

Energy storage devices (ESD) which are intended to power electronic devices, used in close contact of human skin, are desirable to be safe and non-toxic. In light of this requirement, Zn based energy storage devices seem to provide a viable pathway as they mostly employ aqueous based electrolytes which are safe and non-toxic in their functioning. Additionally, having a flexible ESD will play a crucial role as it will enable the ESD to conform to the varying shapes and sizes of wearable electronics which they energize. In this work, we have developed an inkjet-printed Zinc ion battery (IPZIB) with planar electrode configuration over bond paper substrate. Zn has been used as the negative electrode, MnO_2_ is used as the positive electrode with Poly(3,4-ethylenedioxythiophene) polystyrene sulfonate (PEDOT:PSS) as the active binder. Conducting tracks of reduced graphene oxide (rGO) are used to construct the current collector on the paper substrate. The fabricated IPZIB delivered a high discharge capacity of 300.14 mAh g^−1^ at a current density of 200 mA g^−1^. The energy density of the IPZIB is observed as 330.15 Wh kg^−1^ at a power density of 220 W kg^−1^ and retains an energy density of 94.36 Wh kg^−1^ at a high power density of 1650 W kg^−1^. Finally, we have demonstrated the capability of the IPZIB to power a LED at various bending and folding conditions which indicates its potential to be used in the next generation flexible and wearable electronic devices.

## Introduction

Energy and its optimal utilization serve as the foundational pillar and primary driver of technological evolution. Renewable sources of energy such as solar, wind and hydro are constrained by their intermittent nature dependent on weather patterns and diurnal cycles. The unforeseen gap between energy supply and demand can be bridged by energy storage systems^1–3^. Therefore, there is a need for energy storage systems characterized by exceptional electrochemical performance, eco-friendliness, widespread accessibility, affordability, and enhanced safety profile. Energy storage materials that are widely reported considering non-toxicity, abundance and intrinsic safety level include Na, K, Mg, Ca, Al, and Zn^4–8^. Zn offers a good overall compatibility in humid atmosphere with a comparatively higher theoretical capacity than other aqueous electrolyte-based compatible anodic materials^9–11^. Furthermore, zinc can be directly used as anode in aqueous electrolyte unlike other metals whose electrochemical redox voltages fall beyond the stable potential window of water^12–14^. The Cathode materials that are mainly reported in Zinc-ion batteries (ZIBs) include derivatives of Prussian blue, Vanadium, Manganese and quinone based compounds^15–18^. Owing to the high theoretical capacity, nontoxicity, low cost and high overall voltage, Mn-based cathode materials seem to be a suitable cathode material for ZIBs. However, manganese dissolution and fading capacity is a major issue^19–21^. Manganese dioxides occur in various crystallographic forms such as α-MnO_2_ (hollandite), β-MnO_2_ (pyrolusite), γ-MnO_2_ (nsutite), δ-MnO_2_ (birnessite) and λ-MnO_2_ (spinel), each of these crystal structure has a direct influence on the charge storage capacity and reversibility^22^. δ-MnO_2_ which has a layered crystal structure, facilitates intercalation/de-intercalation of ions, and is found to show superior electrochemical performance^23–25^.

MnO_2_ has a very low electrical conductivity which demands the usage of conductive additives such as carbon black, carbon nanotubes, graphene, and other conductive carbonaceous materials^26–28^. In addition to a conductive additive, a binder is also needed to maintain the mechanical integrity and adhesion of the electrode material to the substrate. The binders that are generally used are insulating and electrochemically inactive which results in dead mass reducing the specific capacity of the electrode. A special class of polymers known as the conducting polymers are reported to serve the purpose of being both electrochemically active and binding in nature as regards the active materials put together^29^. Poly(3,4-ethylenedioxythiophene) polystyrene sulfonate (PEDOT:PSS) is a conducting polymer that has been reportedly used as an active conductive binder in various ESD applications^30–33^.

The electrode configuration that are mostly reported for ZIBs are planar and sandwiched types^23,24,34^. Among the two configurations, the planar configuration has certain advantages like exclusion of separators which reduces ion transport resistance, having the electrode and electrolyte on the same plane which reduces thickness rendering it amenable for flexible devices and the planar electrodes can be integrated directly with a circuit pattern in flexible electronics. Secondly, in planar configuration, heat dissipation is more effective owing to the larger exposed surface area of the device. Thus, making it amenable to high power applications where there is higher heat generation. The fabrication techniques that are generally used for ESDs are blade coating, screen printing and electrodeposition. Other techniques like 3D printing and inkjet printing have also been reported^27,34–36^. Inkjet printing has the advantage of digitally designing complex print patterns with fully controllable thickness and shape, size scalability, very low material wastage allowing one to print intricate features that can serve the purpose of both energy storage and aesthetics in wearable electronics^37–39^. However, the major bottleneck of this technique is the formulation of the right ink constitution that is printable and has a long shelf life.

In this work, we have reported an inkjet-printed ZIB (IPZIB) in a planar configuration using bond paper as the substrate. Zn has been used for the negative electrode and a composite of PEDOT:PSS and MnO_2_ has been used for the positive electrode. Reduced graphene oxide (rGO) has been used as the current collector and a polyvinyl alcohol (PVA)-based aqueous gel electrolyte has been used to fabricate the final all solid state IPZIB. A detailed systematic approach for formulation of the various inks with good printability and shelf life has been discussed. The electrochemical properties have been extensively tested at various bending conditions demonstrating the capability of the fabricated device for their direct use in flexible electronics. There are numerous works being carried out in ZIBs focusing mainly on improving performance. As per the literature, this is a first of its kind work which provides a detailed methodology for developing a process for fabricating inkjet-printed ZIB on paper substrate that could find potential applications in flexible electronics.

## Experimental methods

### Materials

Zinc nanoparticles (≥ 99%, 40–60 nm), PEDOT:PSS (1.3 wt% in H_2_O, conductive grade), Xanthan gum, Triton X-100 (Lab grade), Ethanol (AR grade) and acetic acid (99.8%) were purchased from Sigma Aldrich. 2-Butoxyethanol (98%), Manganese sulfate (MnSO_4_⋅H_2_O, 99%), Hydrazine hydrate (80% AR), Diethyl ether (98%), Sodium Nitrate (NaNO_3_, 99%) were all purchased from Loba Chemie. Potassium permanganate (99%), Sulfuric acid (98%), Graphite powder (100 μm) was purchased from S D Fine Chem Limited. N-Methyl-2-pyrrolidone (NMP, 99%) and Hydrogen peroxide (H_2_O_2_, 30% w/w) were purchased from Thermo fisher Scientific. Dipropylene glycol (99%), Polyvinyl butyral (PVB, P 2063) and Carbon black (99.9%) were purchased from Ottokemi.

### Electrode material synthesis and ink formulation

#### Negative electrode ink

The negative electrode is prepared using Zn nanoparticles. Briefly 8 mg/ml of Zn nanoparticles have been added to a solvent mixture. The solvent mixture is prepared by mixing 70% 2-Butoxyethanol, 20% dipropylene glycol and 10% distilled water by volume. 1 mg/ml of PVB is further added and the solvent mixture is stirred for 30 min. The prepared ink is then set for bath sonication for 1 h followed by probe sonication for 30 min. External cooling by ice is done while probe sonication to control the rise in temperature. The sonicated ink is then passed through a 1 μm syringe filter to exclude the agglomerated large sized particles.

#### Positive electrode ink

A composite of PEDOT:PSS and MnO_2_ (PEDOT:PSS-MnO_2_) has been used as the positive electrode. MnO_2_ is synthesized by a hydrothermal process. 0.2 g MnSO_4_⋅H_2_O and 0.4 g KMnO_4_ are mixed in 80 ml distilled water. The solution is stirred for 10 min and then transferred to a 100 ml Teflon-lined autoclave and maintained at 160 °C for 6 h followed by natural cooling to room temperature. The obtained material is then centrifuged, washed with deionized water several times, and dried at 50 °C in a vacuum oven. For the ink preparation, 10 mg/ml of MnO_2_ and 1 mg/ml of carbon black are added to the solvent mixture. The solvent mixture is prepared by mixing 60% N-Methyl-2-Pyrrolidone, 20% PEDOT:PSS and 20% ethylene glycol by volume. The solvent mixture for the positive electrode ink without PEDOT:PSS is prepared by replacing 20% PEDOT:PSS with 20% distilled water by volume and 1 mg/ml of PVB. The prepared ink is then bath sonicated and filtered in the same manner as before.

#### Current collector ink

Reduced graphene oxide (rGO) is used as the current collector material owing to its exceptional properties like high electrical conductivity, chemical stability, mechanical strength and flexibility. Graphene oxide (GO) is synthesized by an improved version of the Hummer’s method reported previously^40^. Briefly, KMnO_4_ is added in small portions to a mixture of H_2_SO_4_, H_3_PO_4_ and graphite flakes while maintaining the temperature below 40 °C. The reaction is then heated and stirred for 12 h and then cooled to room temperature followed by pouring it into a mixture of ice and H_2_O_2_. The solution was then subjected to multiple filtration process. Finally, the remaining material was coagulated with ether and extracted by membrane filtration followed by vacuum drying. The synthesized graphene oxide is then reduced by hydrazine hydrate according to a previously reported work^41^. The ink is prepared by making an 8 mg/ml solution of the synthesized rGO in a solvent mixture of 60% N-Methyl-2-pyrrolidone, 20% distilled water and 20% ethylene glycol by volume. 1 mg/ml of PVB was then added to the solvent mixture. The prepared ink is bath sonicated and filtered in the same manner as before.

#### Curing ink

The curing ink is prepared according to a previously reported work^42^. Briefly 8 wt% of acetic acid is added in a solvent mixture. The solvent mixture is prepared by adding 0.005 wt% Triton X-100 and 0.001 wt% Xanthan gum to a solution of 1% 2-Butoxyethanol and 99% distilled water by volume. The prepared ink is then bath sonicated for 1 h.

### Material characterization

The morphological characterization of the synthesized material is done through field emission scanning electron microscopy (Nova NanoSEM 450) and transmission electron microscopy (FEI-Titan G2 60–300 kV TEM). Elemental analysis is done by energy dispersive x-ray spectroscopy (EDX) using the Nova NanoSEM 450 module equipped with a Bruker SDD-EDS detector. The crystal structure characterization of the synthesized material is done by X-ray diffraction (Panalytical XRD) using Cu K-α radiation from 5° to 70° at a scan rate of 2° min^−1^. Raman spectroscopy (Princeton Instruments Acton Spectra Pro 2500i) is performed using a 532 nm DPSS laser in the range of 200–2000 cm^−1^. The particle size distribution of all inks is ascertained using dynamic light scattering (DLS) particle analyzer (Beckman Coulter Delsa Nano C). The viscosity of all inks is measured using a rheometer (Anton Paar) and the surface tension is measured through a contact angle goniometer (Krüss DSA 25).

### Printing of the electrodes

**Figure 1:**
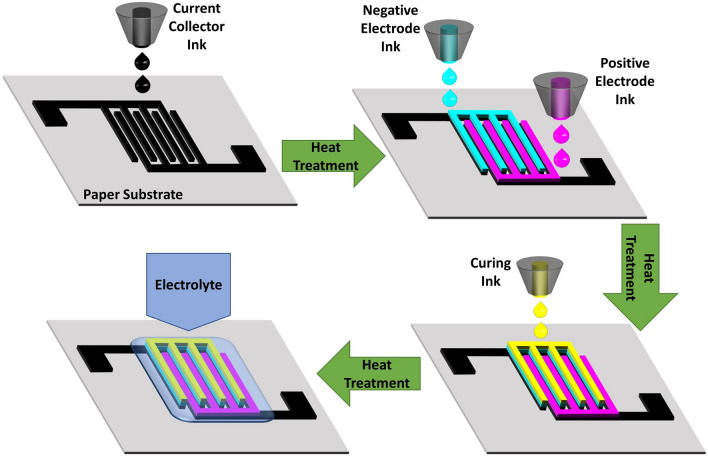
A schematic showing the various steps involved in fabrication of the IPZIB.

The printing of the electrodes is carried out by using an inexpensive inkjet printer Epson L130. Prior to printing, all the different inks have been optimized by varying the material loading and solvent ratio so that the physical properties of the inks possess an inverse Ohnesorge number (Oh^−1^ or Z) value in permissible range, i.e., 1 < Z < 10. The four inks namely the negative electrode, positive electrode, current collector, and the curing ink had been filled separately in four clean ink tanks of the printer used for dispensing the cyan, magenta, black and yellow colors, respectively. The colour of each electrode was changed accordingly so that the desired ink could be drawn from the specific colour ink tank. The printing was done in high quality mode to maximize the material deposition and resolution. The substrate used for printing was 100 GSM A4 size bond paper. The steps for the printing process are shown schematically in Fig. 1. Initially, conductive tracks of rGO were printed on the bond paper which served as the current collector. After 11 layers of printing, the printed paper is heat treated at 70 °C for 12 h in a vacuum oven. The number of printing layers for the positive and the negative electrodes were 6 and 3 layers, respectively which are based on the theoretical specific capacity of each electrode material so that the charge storage is equalized. The printed electrodes are heat treated as done previously. Finally, a single layer of the curing ink is printed over the negative electrode and heat treated as before. The digital pattern, color sequence and the various dimensions used for printing the IPZIB are provided in Fig. S1. The active area of the IPZIB is measured to be approximately 3.13 cm^−2^ with an areal mass density of 6.8 mg cm^−2^.

### Fabrication of the complete battery

The final device is fabricated by drop casting a gel electrolyte over the already printed inter-digitated electrode pattern as shown in Fig. 1. The gel electrolyte is prepared by mixing 3 M ZnSO_4_, 0.5 M MnSO_4_^24^ to suppress Mn^2+^ dissolution and 3 g polyvinyl alcohol (PVA) in 100 ml distilled water and stirred for 2 h at 80 °C. This drop-casted device is then dried at 45 °C for 12 h to remove the excessive moisture and enhance the gelling of the electrolyte. Finally, it is packaged using a parafilm roll and sealed with a cellophane tape.

### Electrical and electrochemical measurements

The sheet resistance of the printed patterns is measured using a sheet resistance measurement system (Pro4). Electrochemical performance is analyzed by cyclic voltammetry (CV), galvanostatic charge discharge (GCD), and electrochemical impedance spectroscopy (EIS) using an electrochemical workstation (Metrohm Autolab Multichannel Potentiostat/Galvanostat M204) at room temperature. The complete battery is tested in a two-electrode setup. Cu tapes are used for connections. The electrochemical impedance spectroscopy (EIS) measurements are carried out using a 5 mV AC amplitude in a frequency range of 1 MHz to 100 mHz at open circuit potential.

## Results and discussion

### Materials characterization

**Figure 2:**
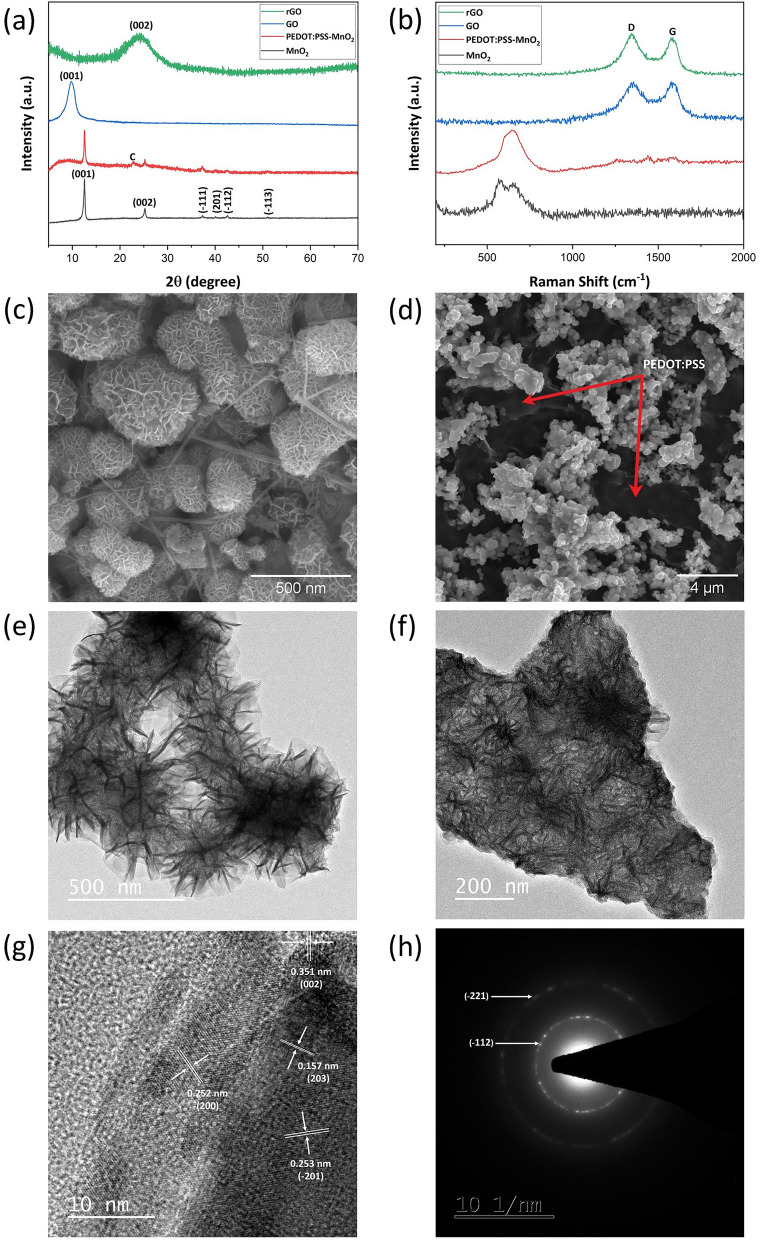
(**a**) XRD patterns of the synthesized MnO2, PEDOT:PSS-MnO_2_, GO and rGO. (**b**) Raman spectra of the synthesized MnO2, PEDOT:PSS-MnO_2_, GO and rGO. (**c**) SEM image of the synthesized MnO_2_. (**d**) SEM image of the PEDOT:PSS-MnO_2_ composite. (**e**) TEM image of the synthesized MnO_2_. (**f**) TEM image of the PEDOT:PSS-MnO_2_ composite. (**g**) HRTEM image of PEDOT:PSS-MnO_2_ composite. (**h**) SAED pattern of the PEDOT:PSS-MnO_2_ composite.

Figure 2a shows the XRD patterns of MnO_2_, PEDOT:PSS-MnO_2_, GO and rGO. The XRD pattern of the synthesized MnO_2_ matches very well with the birnessite phase of potassium manganese oxide hydrate or δ-MnO_2_ (ICDD reference code: 01-080-1098) which possesses a layered crystallographic structure that facilitates intercalation and de-intercalation of foreign cations during charge and discharge cycles. The presence of all the peaks implies the successful synthesis of δ-MnO_2_ with high purity. The XRD pattern of the PEDOT:PSS-MnO_2_ composite retain all the major peaks of the synthesized MnO_2_ indicating no structural changes in MnO_2_ during the composite ink formulation. An additional peak at 22.74° appear which correspond to the presence of carbon black. The XRD pattern of GO shows a peak at a 2θ value of 9.73° which correspond to the (001) plane. Reduction of GO to rGO result in disappearance of the peak at 2θ = 9.73° and appearance of a broad peak at 2θ = 24.19° corresponding to (002) plane. This might be due to partial restacking of the oxidized graphene layers. Figure 2b shows the Raman spectra of MnO_2_, PEDOT:PSS-MnO_2_, GO and rGO. The Raman spectrum of MnO_2_ show two prominent peaks centred around 567 and 650 cm^−1^ which correspond to the symmetric Mn–O stretching vibration in the basal plane of MnO_6_ octahedron^43,44^. The Raman spectrum of PEDOT:PSS is given in Fig. S2b which show a broad peak ranging from 1490 to 1534 cm^−1^ corresponding to various types of C=C bond vibrations^45^. In the PEDOT:PSS-MnO_2_ composite, the two peaks for MnO_2_ are seen to have merged into a single peak centred at 649 cm^−1^ and a series of minor peaks from 1260  to  1606 cm^−1^ have been seen which possibly correspond to the PEDOT:PSS. For GO and rGO, the two peaks at 1350 cm^−1^ and 1585 cm^−1^ correspond to the lattice distortion due to D and G mode of vibration respectively.

The synthesized MnO_2_ is found to have a nanoflower-like form with sizes ranging from 50 to 500 nm. This morphology is desirable as it will have more contact area with the electrolyte and facilitate more pathways for ion transfer (Fig. 2c). The SEM images of the PEDOT:PSSS-MnO_2_ composite is shown in Fig. 2d. It can be seen that the MnO_2_ is fully covered in the matrix of PEDOT:PSS. This is favourable for the flow of electrons generated by MnO_2_ during the electrochemical reactions by the conductive PEDOT:PSS matrix. TEM images of the synthesized MnO_2_ and the PEDOT:PSS-MnO_2_ composite also showed the same morphology like the SEM images (Fig. 2e and f). The high-resolution transmission electron microscopy (HRTEM) image shows the lattice spacings of 0.351, 0.253, 0.252 and 0.157 nm corresponding to the interplanar distance for the (002), (− 201), (200) and (203) crystal planes of synthesized MnO_2_ (Fig. 2g). The SAED pattern obtained shows polycrystalline nature of the composite and the diffraction rings correspond to the (−112) and (−221) plane of MnO_2_ (Fig. 2h). A TEM image of the rGO sheet is shown in Fig. S3. All these findings are quite consistent with the XRD data. Therefore, it can be said that the synthesized MnO_2_ has a layered structure with a nanoflower-like morphology that is beneficial for intercalation/de-intercalation charge storage mechanism with more pathways for the ion movement.

### Printing

For an ink to be printable, physical properties of the ink namely density, viscosity and the surface tension must be in a certain range. This range is determined by a dimensionless number called the Ohnesorge number ($Oh$). The Ohnesorge number is given by:

1 $$Oh=\frac{\sqrt{We}}{Re}=\frac{\eta }{\sqrt{a\rho \gamma }}$$

where $Re=(\rho va)/\eta$ is the Reynolds number and $We=({\rho v}^{2}a)/\gamma$ is the Weber number, $\rho$ is the density, $\eta$ is the dynamic viscosity and $\gamma$ is the surface tension of the ink, $v$ denotes the average ink velocity and $a$ is the characteristic length which is the diameter of the printing nozzle in this case. Another parameter called the Fromm number ($Z={Oh}^{-1}$) is mainly used for defining the printable range for an ink. The range has been updated from time to time and the suggested range for $Z$ by Reis and Derby is $1<Z<10$ for good printability^46,47^. In this case, the printhead nozzle’s diameter is fixed (~ 20 μm) and we are left out with the density, viscosity, and surface tension modulation of the ink which can make the ink printable. Co-solvents, surfactant and binders are mainly used in ink formulation. The viscosity is adjusted by the co-solvent, surfactant controls the surface tension of the ink which is necessary to reduce the Marangoni effect and binders are used to ensure structural integrity and good adhesion of the ink to the substrate.

**Figure 3:**
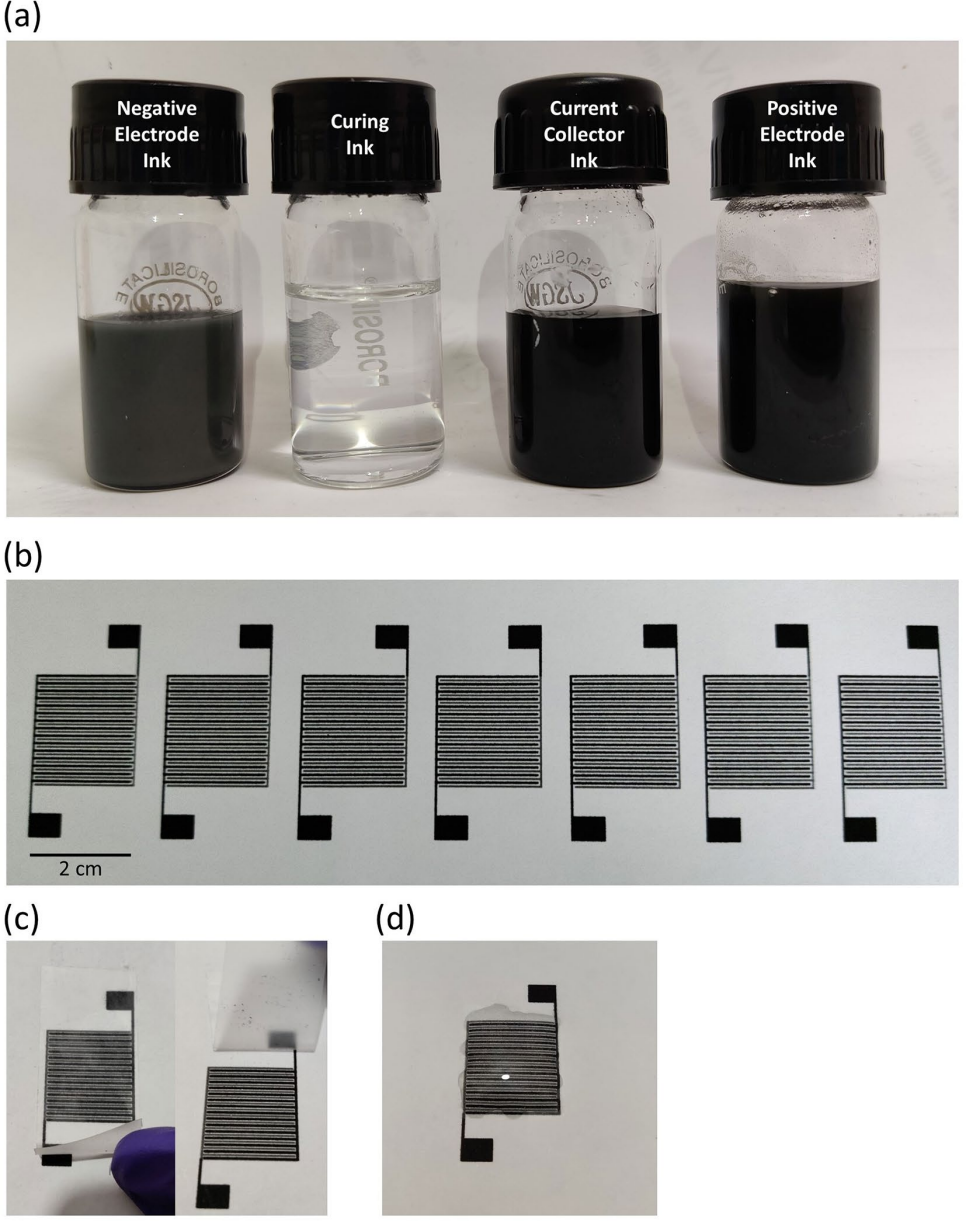
(**a**) A photograph of the the negative electrode, curing, current collector and positive electrode ink. (**b**) Bulk printing of 7 IDE pattern in a row for the IPZIB. (**c**) Adhesive tape test of the printed electrodes. (**d**) Drop casting of the gel electrolyte over the printed electrodes.

The negative electrode ink was formulated using 2-Butoxyethanol as the main solvent, dipropylene glycol as the co-solvent, water as an agent to increase surface tension and polyvinyl butyral as the binder material. The change in viscosity of the negative electrode ink with co-solvent and Zn concentration is shown in Fig. S4a and b. The change in surface tension with water is shown in Fig. S4c. A volumetric percentage of 25% for co-solvent and 5% for distilled water with a Zn concentration of 8 mg/ml gives us a suitable value of $Z$ = 5.26. The positive electrode ink is formulated using N-Methyl-2-Pyrrolidone as the main solvent, ethylene glycol as the co-solvent and PEDOT:PSS as the conductive binder. The change in viscosity with co-solvent and MnO_2_ concentration is shown in Fig. S5a and b. A volumetric percentage of 20% ethylene glycol, 20% PEDOT:PSS and 10 mg/ml of MnO_2_ results in a $Z$ value of 5.95 which is in the printable range. The current collector ink is formulated using the same main solvent and co-solvent that is used for the positive electrode ink. A volumetric percentage of 20% ethylene glycol, 20% distilled water and 8 mg/ml of rGO result in a $Z$ value of 5.78 which is again in the printable range. The curing ink is formulated based on a previously reported work. The acetic acid concentration in the curing ink is changed and optimized for the best electrochemical performance of the negative electrode. An 8 wt% concentration of acetic acid is found to give the best result. The optimization of the acetic acid concentration is discussed in later sections. The curing ink possesses a $Z$ value of 5.97 which is in the printable range. The viscosities of all the inks are measured at a shear rate of 300 s^−1^ at room temperature. The density, viscosity, surface tension and the Fromm number of all the prepared ink is given in Table S1. In inkjet printer, the ink is subjected to very high shear rate at the nozzle end. In such a case, a non-Newtonian fluid will undergo significant change in the viscosity and the formulated ink comes out of the printable range as per the Fromm number condition. Thus, the change in viscosity of the ink with the shear rate must be further studied. Figure S7 shows the variation of the dynamic viscosity of all the inks with their shear rates. It can be clearly seen that the viscosity stabilizes after a shear rate of 100 s^−1^ and remains nearly constant up to shear rate of 10^4^ s^−1^ for all the inks indicating that their behaviour is by and large Newtonian. Thus, it can be speculated that the ink may maintain a constant viscosity and remain printable when subjected to the high shear rate in the inkjet printer. The DLS results for all the inks are shown in Fig. S8. It is found that the particle size for all the formulated inks is within 1 μm which ensures that issues of nozzle clogging should not arise. Figure 3a shows an image of the different inks used in the fabrication of the IPZIB.

The current collector ink is used to print the conductive tracks on the paper substrate. The change in the sheet resistance with the number of printed layers is shown in Fig. S9a. The sheet resistance with a single printed layer of the current collector ink is found to be 1693.4 Ω sq^−1^ which drops down to a value of 6.91 Ω sq^−1^ with 11 printed layers and then remains almost constant. The cross-sectional thickness of the current collector with 11 printed layers is measured as approximately 10 μm as shown in Fig. S9b. Apart from the number of printed layers, the post heat treatment of the printed layers is also crucial as it removes all the solvent used in the ink formulation process which may otherwise hinder the electrical performance and film continuity. The temperature for heat treatment must be selected such that it is below the melting point of the binders used in the ink formulation otherwise adhesion of the ink materials to the substrate may start deteriorating and it may also lead to thermal damage of the substrate. Therefore, mild heating in vacuum with longer duration is preferable. The theoretical specific capacity of Zn is 820 mAh g^−1^ and MnO_2_ is 308 mAh g^−1^^48,49^_._ The concentration of Zn in the negative electrode ink is 8 mg/ml and MnO_2_ in the positive electrode ink is 10 mg/ml. Based on Eq. (S4), the desired ratio of printing layers for positive electrode to the negative electrode is calculated to be 2. 3 printed layers of the negative electrode and 6 layers for the positive electrode are found to give the highest specific discharge capacity and this configuration is used for further study (Table S2). An optimization of the thickness of the electrode is needed because the charge storage in the cathode occurs by surface adsorption and intercalation of ions. When we increase the cathode thickness beyond an optimum level, the ions cannot intercalate into the larger depths of the cathode rendering the innermost part of the cathode ineffective which results in decrease of the specific discharge capacity.

After the printing of current collector, positive and negative electrode, a layer of curing ink is printed over the negative electrode. The printing of the curing ink is an important step to activate the negative electrode. Zn being the active material in the negative electrode undergoes spontaneous oxidation and forms a thin layer of ZnO and Zn(OH)_2_ on the Zn nanoparticle surface^50,51^. This passivating layer of ZnO and Zn(OH)_2_ hinders the electrical and electrochemical performance of the electrode. Weak acidic solutions using acetic acid have been reported to dissolve this passivation layer and form interconnects between the Zn nanoparticles which result in improved electrical and electrochemical performance^52,53^. A more comprehensive explanation of this phenomenon can be found in the work presented by Jayasayee et al.^53^. Majee et al. has also reported a single layer of 3–10 wt% of acetic acid concentration in a 1:100 volume of ethylene glycol butyl ether and water-based curing ink to give the best electrical performance^42^. In this work, a concentration of 5, 8 and 12 wt% of acetic acid has been used and a concentration of 8 wt% acetic acid has been found to give the lowest sheet resistance of 4.78 Ω sq^−1^ for the printed Zn electrode (Table S3). Figure S10 shows the SEM images of the uncured and 8 wt% acetic acid cured Zn electrode. The formation of the interconnects between the Zn nanoparticles can be clearly seen in the cured Zn electrode as reported by other researchers. The EDX results show the compositional difference between the cured and the uncured Zn electrode which further confirms the dissolution of the passivation layer and exposure of the underlying Zn surface (Fig. S11). Figure 3b shows the feasibility of the process for bulk fabrication of printed energy storage devices. The adhesion of the printed materials to the paper substrate is qualitatively assessed by adhesive tapes (Fig. 3c). Finally, a layer of the PVA-based gel electrolyte is drop casted over the printed electrode completing the fabrication of the IPZIB (Fig. 3d).

### Electrochemical performance

**Figure 4:**
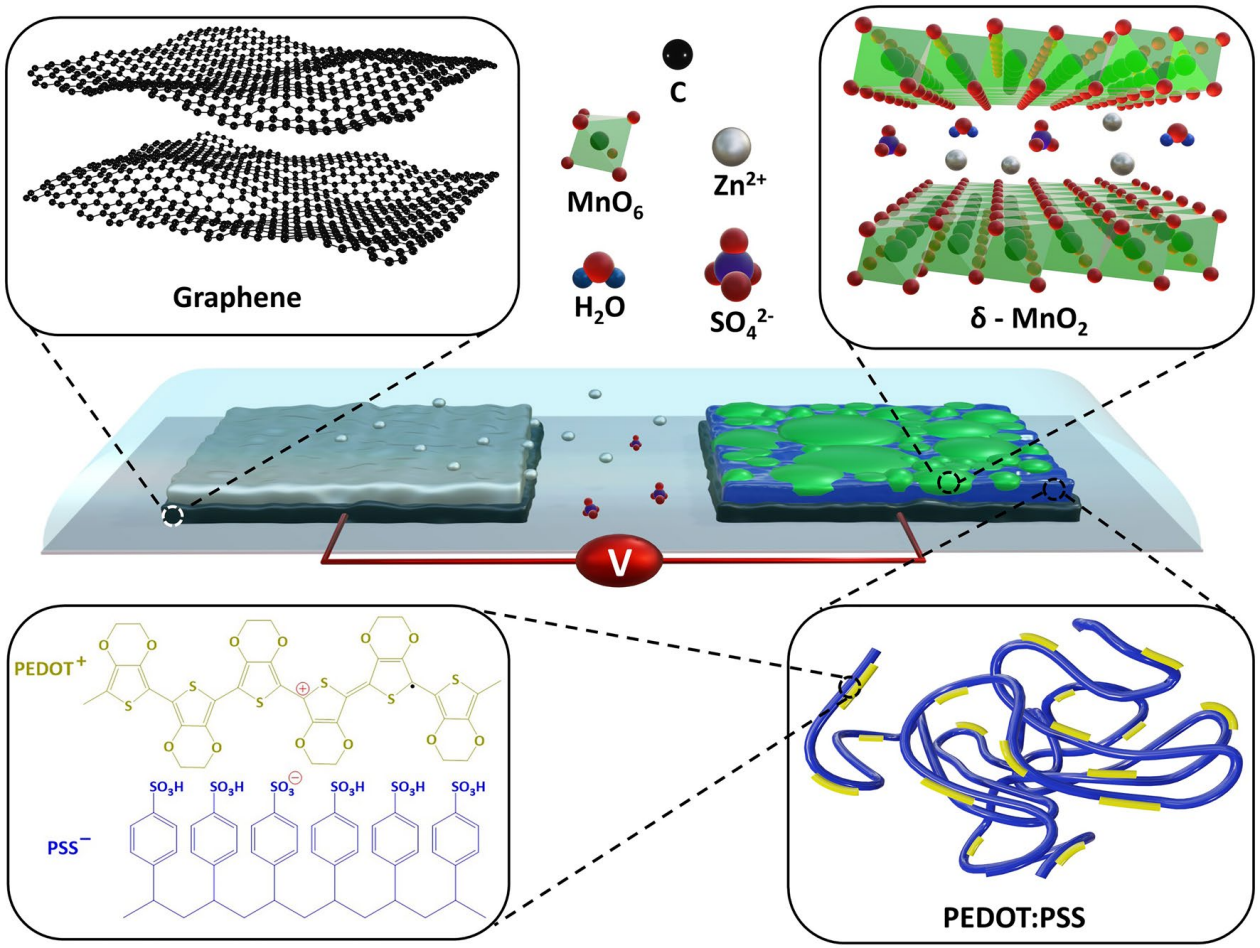
A schematic of the IPZIB setup showing the various components. The inset shows a model of the rGO sheets, the layered structure of δ-MnO_2_, the PEDOT:PSS polymer chains and the chemical structure of PEDOT:PSS. The objects used in the figure are not to scale.

A schematic of the IPZIB setup with the various components is shown in Fig. 4. The high electrochemical performance of δ-MnO_2_ is mainly attributed to its layered crystallographic structure which facilitates intercalation/de-intercalation mechanism of reaction for cations. The electrochemical reactions at the two electrodes can be given as follows^24^:

At negative electrode:

2 $${\text{Zn}} \rightleftharpoons {{\text{Zn}}}^{2+}+2{e}^{-}$$

At positive electrode:

3 $${\text{MnO}}_{2}+{{\text{H}}}^{+}+{e}^{-}\rightleftharpoons {\text{MnOOH}}$$

4 $$4{\text{MnO}}_{2}+2{{\text{Zn}}}^{2+}+4{e}^{-}+{{\text{H}}}_{2}{\text{O}}\rightleftharpoons {{\text{Zn}}}_{2}{{\text{Mn}}}_{4}{{\text{O}}}_{8} \cdot {{\text{H}}}_{2}{\text{O}}$$

5 $$2{\text{MnOOH}}+{\text{SO}}_{4}{^{2-}}+4{{\text{Zn}}}^{2+}+7{{\text{H}}}_{2}{\text{O}}+2{e}^{-}\rightleftharpoons {{\text{Zn}}}_{4}{\text{SO}}_{4}{\left({\text{OH}}\right)}_{6} \cdot 5{{\text{H}}}_{2}{\text{O}}+ 2{{\text{Mn}}}^{2+}$$

6 $$3{{\text{Zn}}}_{2}{{\text{Mn}}}_{4}{{\text{O}}}_{8} \cdot {{\text{H}}}_{2}{\text{O}}+8{\text{SO}}_{4}{^{2-}}+61{{\text{H}}}_{2}{\text{O}}+26{{\text{Zn}}}^{2+}+12{e}^{-}\rightleftharpoons 8{{\text{Zn}}}_{4}{{\text{SO}}}_{4}{({\text{OH}})}_{6} \cdot 5{{\text{H}}}_{2}{\text{O}}+12{{\text{Mn}}}^{2+}$$

During discharge reaction, Zn is oxidized to Zn^2+^ at the negative electrode as given by Eq. (2). At the positive electrode, there are a series of reactions occurring. Equations (3) and (4) are related to the intercalation of H^+^ and Zn^2+^ ions into the layered MnO_2_ resulting in the reduction of MnO_2_ from Mn^4+^ to Mn^3+^. The products formed by Eqs. (3) and (4) further undergo a conversion reaction to form Zn_4_SO_4_(OH)_6_⋅5H_2_O as given by Eqs. (5) and (6). During these conversion reactions, Mn^3+^ is further reduced to Mn^2+^ and is dissolved in the electrolyte.

**Figure 5:**
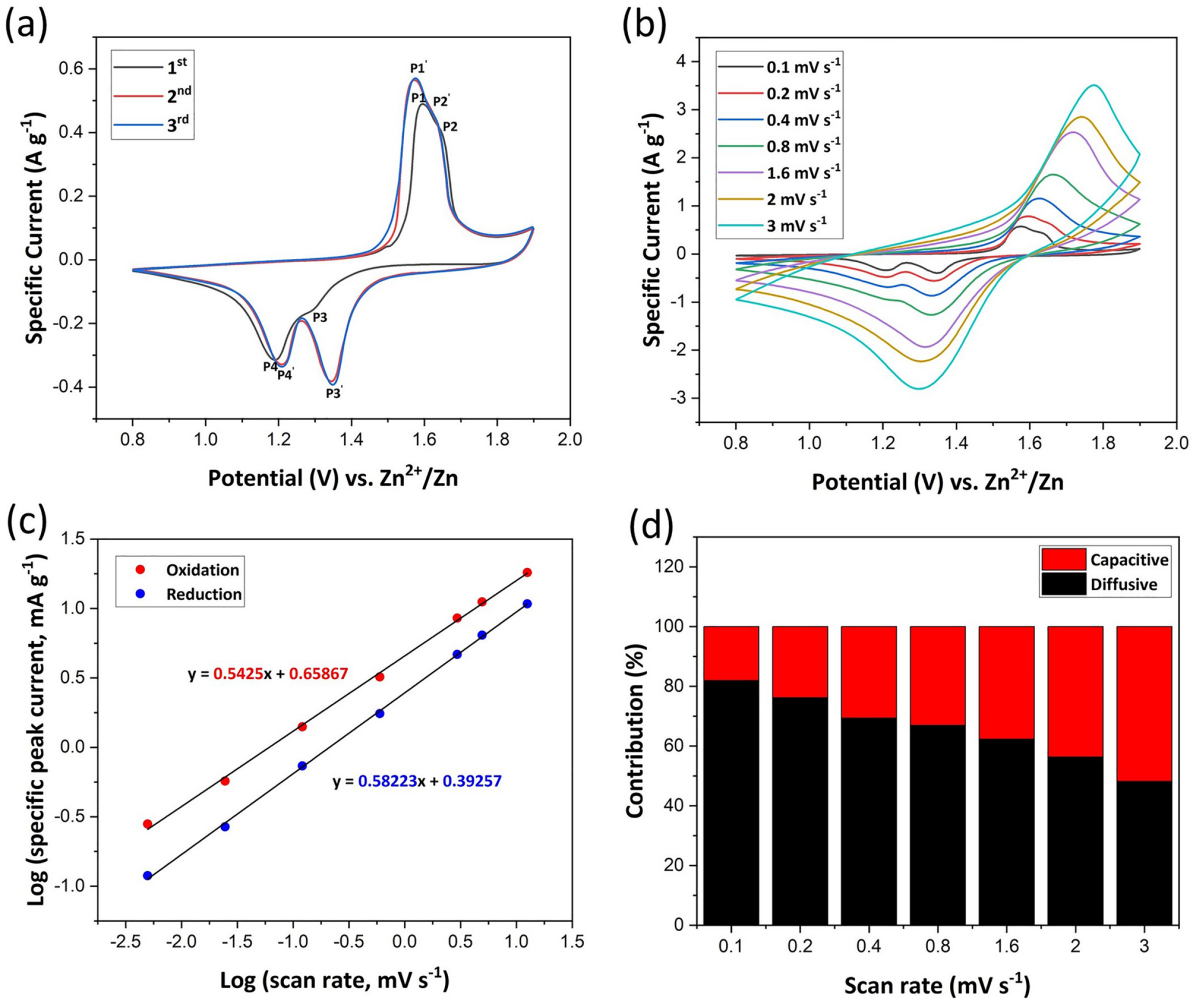
(**a**) The first three CV curve of IPZIB at a scan rate of 0.1 mV s^−1^ in the potential window of 0.8–1.9 V vs. Zn^2+^/Zn. (**b**) The CV curve of IPZIB at different scan rates ranging from 0.1 to 3 mV s^−1^ in the potential window of 0.8–1.9 V vs. Zn^2+^/Zn. (**c**) Linearly fitted ln(peak current) vs. ln(scan rate) corresponding to (**b**). (**d**) Percentage of contribution to charge storage in the IPZIB by capacitive and diffusion controlled mode.

Electrochemical analysis of the IPZIB by CV is shown in Fig. 5. It can be seen in Fig. 5a that in the first cycle there are two convoluted oxidation peaks at 1.59 V and 1.64 V marked as P1 and P2 which then shift to 1.57 V and 1.62 V marked as P1′ and P2′ in the subsequent cycles, respectively. Similarly, there are two reduction peaks at 1.29 V and 1.19 V marked as P3 and P4 which shift to 1.34 V and 1.25 V marked as P3′ and P4′ in the subsequent cycles, respectively. In the first cycle, the peak current value of P3 is low which increases significantly in the subsequent cycles indicating that the reaction occurring at P3 is now contributing more than before. Similarly, the other three peaks are also seen to increase in current intensity in the subsequent cycles which is mainly due to the activation of the electrodes. From the 3rd cycle onwards, the CV curves show almost overlapping properties indicating that the stability of the IPZIB is reached. The intercalation/de-intercalation of Zn^2+^ ions corresponding to Eq. (4) result in the current peaks at P3 or P3′ (intercalation) and P2 or P2′ (de-intercalation). The conversion reaction given by Eqs. (5) and (6) result in the current peak at P4 or P4′ and the reverse reaction result in the current peak at P1 or P1′^24^.

The mode of charge storage in the electrode is further studied by conducting CV at various scan rates in the potential window of 0.8–1.9 V as shown in Fig. 5b. The net charge storage in the electrode can be due to both redox reactions and double layer capacitance. However, the exact mode of charge storage that is contributing more can be discerned by the equation $i=a{v}^{b}$ or ${\text{ln}}i=b ln v+{\text{ln}}a$ , where $i$ denotes the value of the peak current, $v$ is the potential scan rate and $a$, $b$ are fitting parameters^54,55^. The value of $b$ obtained indicate the dominating mode of charge storage. When the value of $b$ is close to 0.5, then the mode of charge storage is mostly diffusional redox reaction and a $b$ value close to 1 indicates capacitive mode of charge storage. As shown in Fig. 5c, the $b$ value for oxidation process is estimated to be 0.54 and for reduction process it comes as 0.58. Furthermore, the percentage contribution from each mode of charge storage is quantified by the Dunn’s method using Eq. (7)^56,57^.

7 $$i\left(V\right)={k}_{1}v+{k}_{2}{v}^{1/2}$$

Here $i\left(V\right)$ is the current value at a fixed potential $V$ which is comprised of a capacitive part (${k}_{1}v$) and diffusion-controlled part (${k}_{2}{v}^{1/2}$). Selecting a potential $V$ and recording the current value $i\left(V\right)$ at each scan rate, can help us plot a graph of $i(V)/{v}^{1/2}$ vs. ${v}^{1/2}$ from which ${k}_{1}$ and ${k}_{2}$ can be determined. Therefore, we can measure the percentage contribution of each mode of charge storage. Figure 5d shows the contribution from each charge storage mode at various scan rates. With increasing scan rate, the percentage contribution from diffusion-controlled mode is seen to reduce which is due to the kinetic limitations of electrochemical redox reactions at higher scan rates.

**Figure 6:**
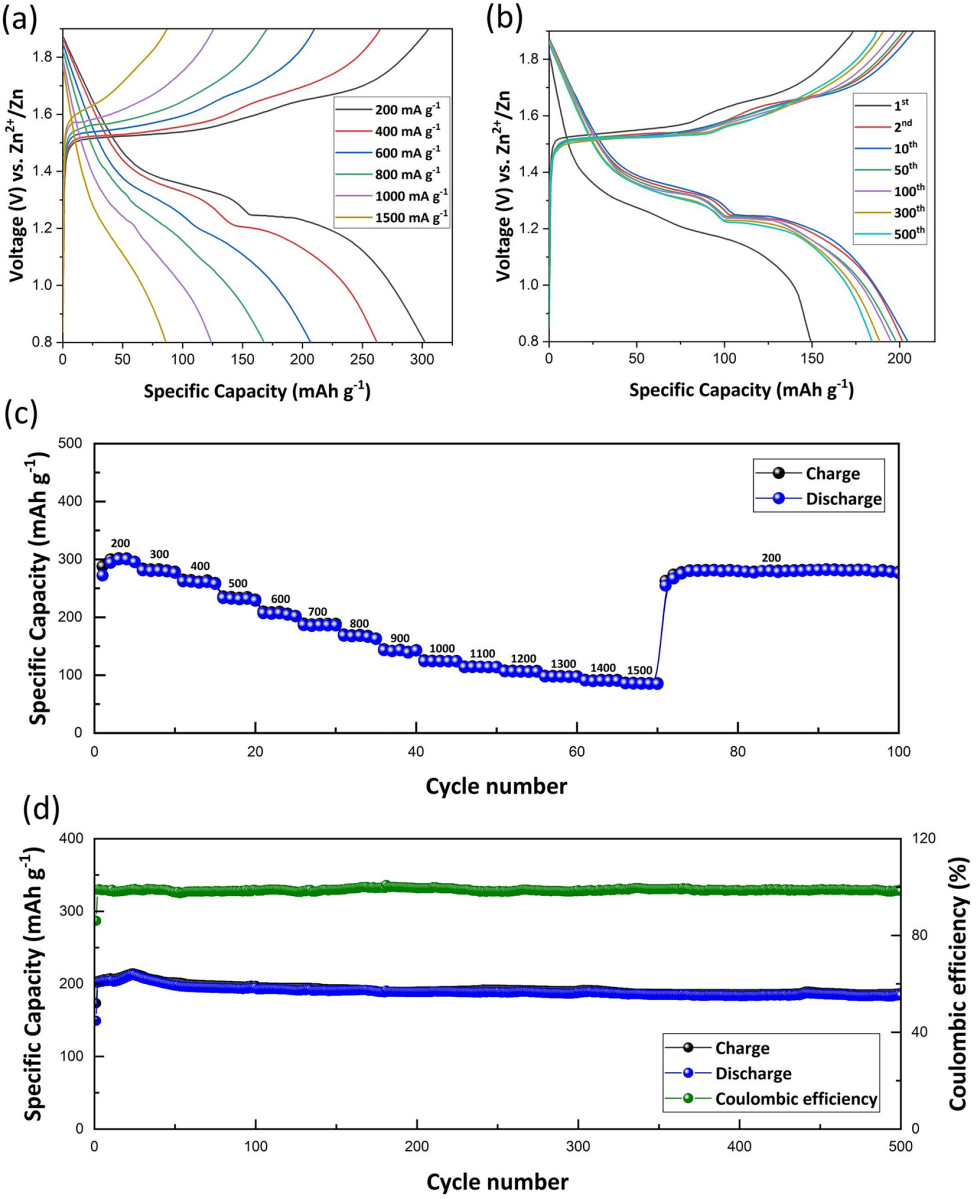
(**a**) Galvanostatic charge/discharge profile of the IPZIB at different current densities in the voltage window of 0.8 to 1.9 V. (**b**) Galvanostatic charge/discharge profile of the IPZIB at a current density of 600 mA g^−1^ for 500 cycles in the voltage window of 0.8 to 1.9 V. (**c**) Rate capability of the IPZIB at different current densities ranging from 200 to 1500 mA g^−1^. (**d**) Long cyclic stability and the coulombic efficiency of the IPZIB corresponding to (**b**).

The electrochemical performance of the IPZIB is further analysed by galvanostatic charge/discharge process. As can be seen in Fig. 6a, the charge/discharge profile of the IPZIB at different current densities in the voltage window of 0.8–1.9 V is shown. The discharge curve has two distinct voltage plateaus up till the discharge current of 600 mA g^−1^. The first plateau during discharge is seen at a voltage of 1.36 V and the second plateau is seen at 1.23 V. Similarly, during charging, there are two plateaus up till 600 mA g^−1^. The first plateau is at 1.52 V and the second one is at 1.66 V. These results are quite consistent with the CV data. With increasing current density, the second discharge plateau becomes less distinct and contributes less to the overall discharge capacity. In the charging curve also, a similar reduction in contribution from the second charge plateau is seen. The rate capability of the IPZIB at current densities ranging between 200 and 1500 mA g^−1^ is shown in Fig. 6c. The IPZIB has a high discharge capacity of 300.14 mAh g^−1^ at a current density of 200 mA g^−1^ and retains a discharge capacity of 124.1 and 85.78 mAh g^−1^ at a current density of 1000 and 1500 mA g^−1^ respectively. This shows that the IPZIB has a good power capability. On decreasing the current density back to 200 mA g^−1^, a high discharge capacity of 281.7 mAh g^−1^ is retained which shows the good stability of the fabricated IPZIB. At each current density, five cycles of charge/discharge are carried out. The average discharge capacity and the corresponding capacity retention at each current density along with the energy and power density is summarized in Table S4. The long cyclic performance of the IPZIB at a current density of 600 mA g^−1^ in the voltage window of 0.8–1.9 V for 500 cycles is also tested. As shown in Fig. 6d, the discharge capacity and the coulombic efficiency is very low in the first cycle which gradually increases and reaches a maximum of 212.345 mAh g^−1^ in the 25th cycle with a coulombic efficiency of 99.24%. The first charge cycle is mainly contributed by the de-intercalation of the already existing K^+^ ions which is then followed by gradual increase in Zn^2+^ intercalation/de-intercalation. The increase in coulombic efficiency also suggests the high reversibility of the charge and discharge reactions. After the 25th cycle, the discharge capacity gradually decreases till 52nd cycle and then remains almost stable with negligible decrease in capacity till the 500th cycle. One reason for such capacity loss is the non-retrieval of all the intercalated Zn^2+^ ions as discussed before. The other major reason is the dissolution of δ-MnO_2_ as Mn^2+^ as given by Eqs. (5) and (6). To supress the dissolution of MnO_2_ and improve the cyclic performance, MnSO_4_ is added in the electrolyte^22^. The movement of these dissolved Mn^2+^ ions is mainly controlled by diffusion. In the absence of MnSO_4_ in the electrolyte, the concentration gradient from the cathode to the electrolyte is high which results in loss of the Mn^2+^ ions to the electrolyte. Therefore, incorporating Mn^2+^ ions in the electrolyte reduces the concentration gradient and suppresses the loss of Mn^2+^ ions to the electrolyte. This results in reduction of the capacity loss of the battery. The charge/discharge profile for the 1st, 2nd, 10th, 50th, 100th, 300th and 500th cycle at 600 mA g^−1^ are shown in Fig. 6b. It can be seen that after the first cycle, the charge/discharge curves for the subsequent cycles are mostly concentrated around each other suggesting good cyclic stability of the fabricated IPZIB. A high discharge capacity of 184.24 mAh g^−1^ is retained after the 500th cycle which is approximately 89% of the discharge capacity in the initial cycles. This high specific discharge capacity and cyclic stability of the IPZIB can be attributed to the presence of MnSO_4_ in the electrolyte which suppresses MnO_2_ dissolution and the presence of PEDOT:PSS as the active binder that further reduces dead mass as will be present in case of other non-conductive binder. An additional specific discharge capacity of 20.51 mAh g^−1^ is achieved by using PEDOT:PSS as the active binder (Fig. S12).

**Figure 7:**
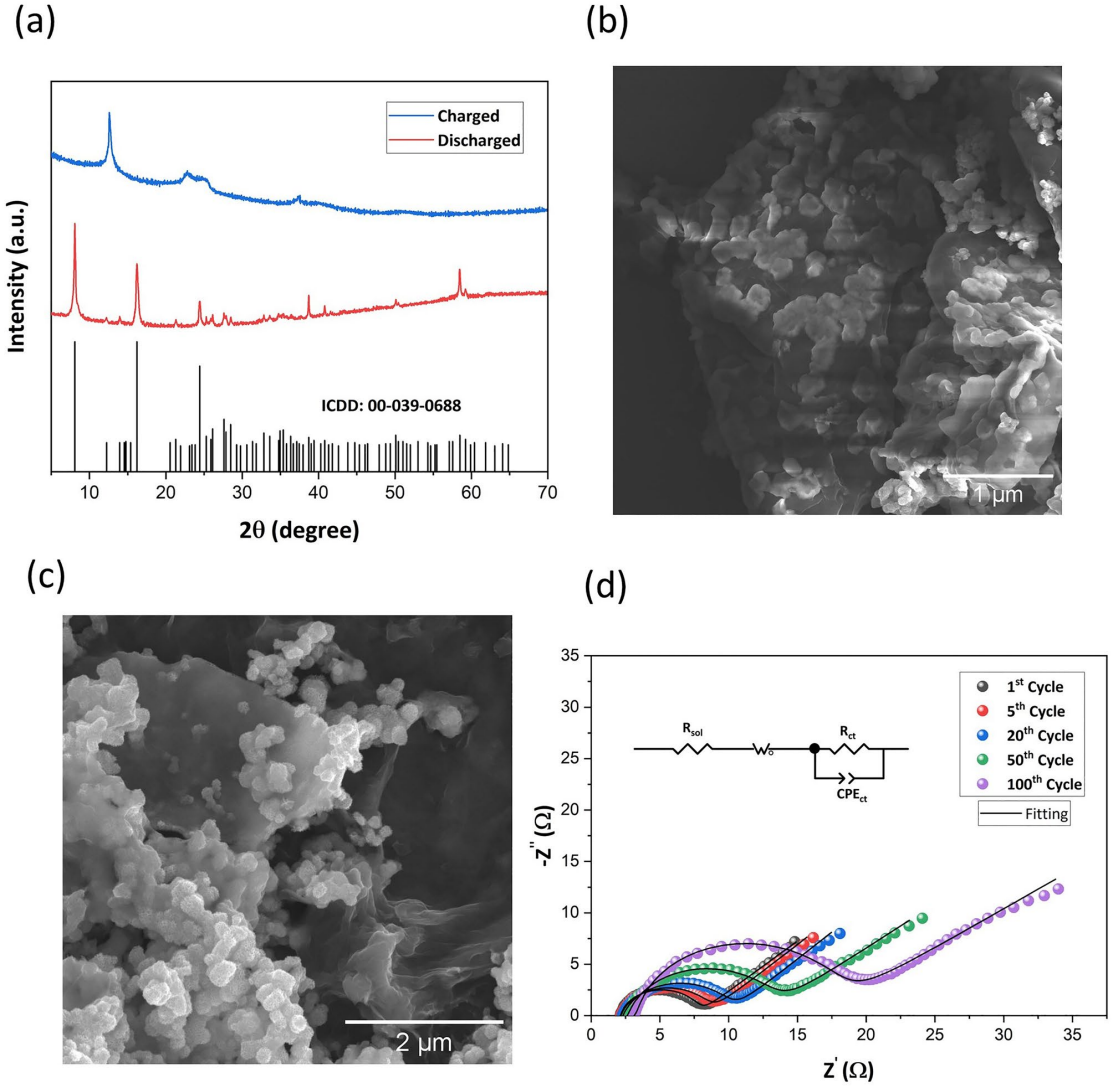
(**a**) XRD patterns of the positive electrode of the IPZIB at discharged (0.8 V) and charged (1.9 V) state. The corresponding SEM image of the positive electrode at (**b**) discharged and (**c**) charged state. (**d**) Nyquist plot of the IPZIB after various cycle of charge–discharge and the corresponding equivalent circuit fitting.

The morphological and crystal structure transformation at the discharged and charged state are further studied using XRD and SEM analysis respectively. Figure 7a shows the XRD patterns of the positive electrode of the IPZIB at discharged and charged state in the 15th discharge and 16th charge cycle at a current density of 200 mA g^−1^. The XRD pattern at the discharged state matches well with XRD pattern of Zn_4_SO_4_(OH)_6_·5H_2_O (ICDD reference code: 00-039-0688). All the major diffraction peaks are present confirming the formation of Zn_4_SO_4_(OH)_6_·5H_2_O as given by the conversion reaction (5) and (6). On charging back to 1.9 V in the 16th cycle, the initial crystal phase of δ-MnO_2_ is seen to have been regained. A slight broadening of the diffraction peaks is seen in the charged state which could be due to reduction in crystallinity of the δ-MnO_2_ phase. This confirms the intercalation/de-intercalation of Zn^2+^ mechanism reaction for charge storage in the fabricated IPZIB as given by Eqs. (3) to (6) with good reversibility. The morphological change of the positive electrode from discharged to charged state is shown in Fig. 7b and c respectively. In the discharged state, a change in the form from nanoflower to flattened button-like form with no petal-like protrusion is seen. These button-like structures are conjectured to be Zn_4_SO_4_(OH)_6_·5H_2_O which is formed in the conversion reaction of the discharge cycle. On charging back to 1.9 V in the 16th cycle, the button-like structure disappears and is transformed back into the nanoflower form confirming that the material regains its initial state indicating good reversibility of the IPZIB. Impedance spectroscopy is a necessary analysis technique for studying the various impedances offered by a device^58–60^. The Nyquist plot of the IPZIB after various cycle of charge–discharge is shown in Fig. 7d and the circuit model used to fit the EIS spectrum consists of a solution resistance (R_sol_), Warburg impedance, charge transfer resistance (R_ct_) and a constant phase element (CPE). The change in the R_sol_ and R_ct_ value after the various cycles of charge–discharge is given in Table S5. Both the R_ct_ and R_sol_ is found to increase after charge–discharge cycling. The increase in R_ct_ can be attributed to both the negative and the positive electrode. The negative electrode undergoes passivation and forms ZnO on the surface on charge–discharge cycling which increases the R_ct_^61^. In the positive electrode, all the intercalated Zn^2+^ ions do not de-intercalate during the charging cycle which then hinder the intercalation of new Zn^2+^ ions. The increase in R_s_ value can be due to loss of moisture from the gel electrolyte as paper is permeable and allows percolation of water from the active area to the other areas of the IPZIB. The slope of the Warburg impedance is found to decrease gradually indicating reduction in the diffusion of ions and this can also be linked to the aforementioned reasons for increase in R_ct_ and R_sol_.

**Figure 8:**
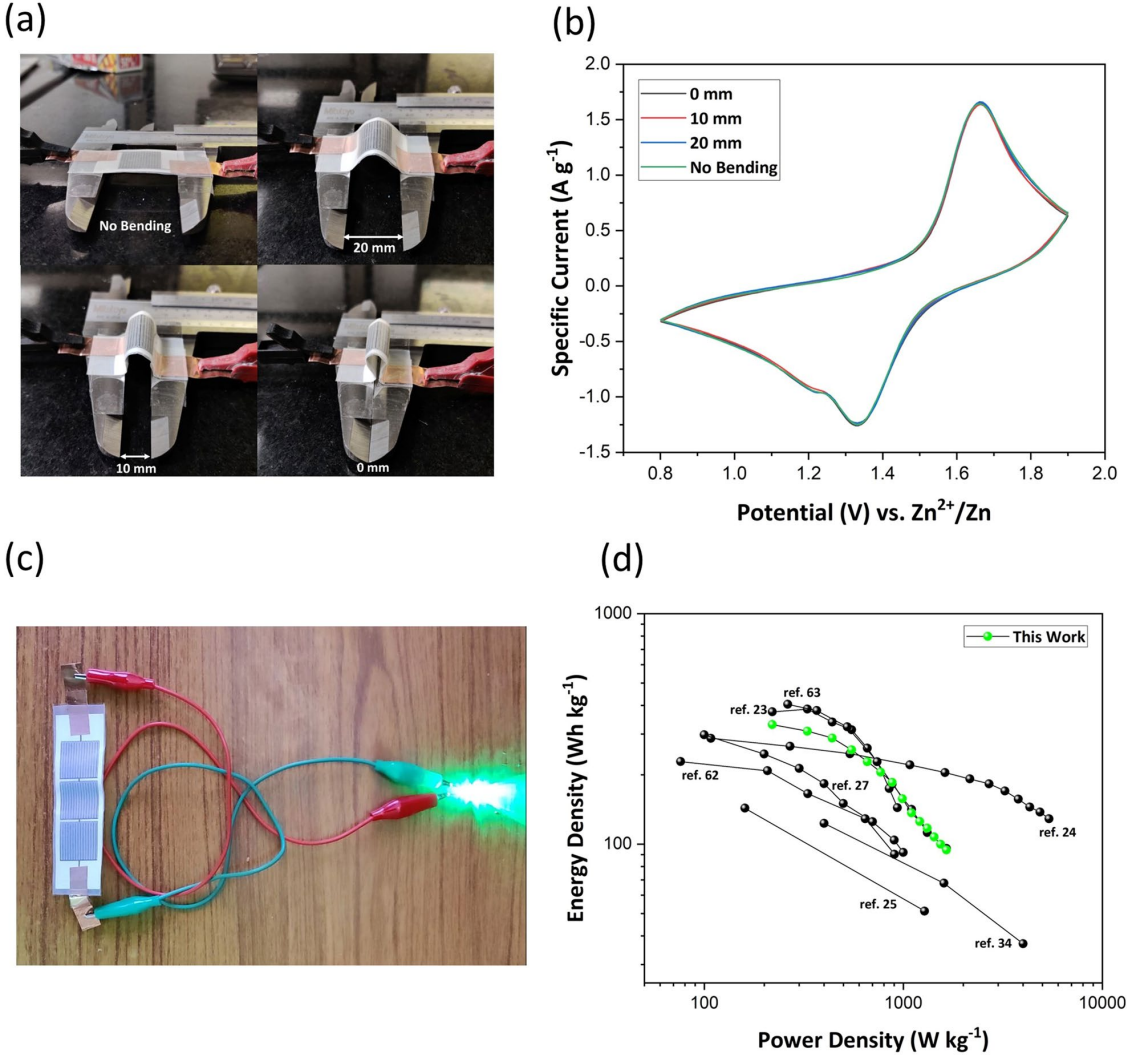
(**a**) Bending of the IPZIB and the corresponding (**b**) CV curves at a scan rate of 0.8 mV s^−1^ in the voltage window of 0.8–1.9 V vs. Zn^2+^/Zn. (**c**) The fabricated IPZIB powering a 3.5 V LED. (**d**) Ragone plot of the fabricated IPZIB and its comparison with other reported high performing ZIBs.

Flexibility of energy storage devices while retaining their electrochemical performance is a prime requirement for efficient utilization in flexible electronics. Under strained conditions, there could be generation of micro-cracks or delamination in the electrode materials which would result in ohmic losses and affect the power capability of the device. Therefore, the fabricated IPZIB is qualitatively assessed under certain bending conditions and the corresponding CV curve is shown in Fig. 8a and b respectively. The CV curves are found to be highly overlapping at all bending conditions indicating good retention of the electrochemical performance. The specific discharge capacity is also measured at the different bending conditions and the capacity retention is nearly 100% (Table S6). This demonstrates the capability of the fabricated IPZIB to be utilized for energy storage in various flexible electronic devices. Finally, we demonstrate the application of the fabricated IPZIB in real-life devices by connecting three cells in series to power a 3.5 V LED (Fig. 8c). This IPZIB can deliver a high energy density of 330.15 Wh kg^−1^ at a power density of 220 W kg^−1^ and an energy density of 94.36 Wh kg^−1^ can be retained at a high power density of 1650 W kg^−1^. A Ragone plot comparing the performance of this IPZIB with various other reported high performing ZIBs is shown in Fig. 8d and it can be seen that the performance of this IPZIB is on par with most of the high performing ZIBs^62,63^.

## Conclusion

In summary, a high performing planar ZIB by inkjet printing technique on bond paper as substrate is shown. Environmentally friendly and non-toxic PVA-based aqueous gel electrolyte is used to fabricate the all-solid state IPZIB. A systematic approach for the ink formulation of the various components of the IPZIB is discussed in detail. The role of the curing ink treatment in activating the Zn electrode is discussed. The presence of PEDOT:PSS as the active binder in the positive electrode contributes to the improved performance of the IPZIB. The intercalation/de-intercalation mode of charge storage in δ-MnO_2_ significantly improves the charge capacity of the IPZIB. The fabricated IPZIB shows a discharge capacity of 300.14 mAh g^−1^ at a current density of 200 mA g^−1^. When cycled at 600 mA g^−1^, the IPZIB retains a high discharge capacity of 184.24 mAh g^−1^ after 500 charge–discharge cycles which is nearly 89% of the initial discharge capacity. The IPZIB delivers a high energy density of 330.15 Wh kg^−1^ at a power density of 220 W kg^−1^ and retains an energy density of 94.36 Wh kg^−1^ at a high power density of 1650 W kg^−1^. Furthermore, the IPZIB is subjected to flexibility test by various bending and folding conditions, and it retains nearly 100% of its capacity at these strained conditions. Therefore, the fabricated IPZIB shows excellent performance and is amenable to be utilized in various sectors like healthcare, sports, military gadgets, soft robotics and IOT for powering the next generation flexible and wearable electronics.

### Supplementary Information

Supplementary Video 1.

Supplementary Information.

## Data Availability

All data analysed during this study are included in this published article and its supplementary information files.
