# Supplementary material for: Inkjet-printed flexible planar Zn-MnO2 battery on paper substrate
Source: Sci Rep. 2024 Jan 18;14:1597. doi: 10.1038/s41598-024-51871-5 (PMC10796916; doi:10.1038/s41598-024-51871-5)
Supplement: Supplementary file 2 — Supplementary Information. [file 41598_2024_51871_MOESM2_ESM.pdf]

Supporting Information

Inkjet-printed Flexible Planar Zn-MnO_2 Battery on Paper Substrate

Sagnik Sarma Choudhury[a,b], Nitish Katiyar[a,b], Ranamay Saha[a,b], and Shantanu Bhattacharya[a,b,*]

a. Department of Mechanical Engineering, Indian Institute of Technology, Kanpur – 208016, India
b. Microsystems Fabrication Laboratory, Indian Institute of Technology, Kanpur – 208016, India
*Corresponding author's Email Id: bhattacs@iitk.ac.in

Electrochemical performance calculations:
The specific discharge capacity was measured from the galvanostatic charge-discharge curve (GCD) according to the following equation:

$$C = \frac{It}{m} \quad \textbf{(S1)}$$

Where C is the specific discharge capacity (mAh g^{-1}), I is the discharge current (mA), t is the discharge duration (h) and m is the mass of the IPZIB.

The specific energy density and the power density was measured according to the following equations:

$$E = C\Delta V \quad \textbf{(S2)}$$

$$P = \frac{E}{t} \quad \textbf{(S3)}$$

Where E is the specific energy density (Wh kg^{-1}), P is the specific power density (W kg^{-1}) and ΔV is the applied voltage window (V).

Calculation of number of print layers for positive and negative electrode:
For the two electrodes to store equal charge, the following equation must be satisfied.

$$n_N \times V_N \times x_N \times C_N = n_P \times V_P \times x_P \times C_P \quad \textbf{(S4)}$$

Where n_N & n_P denote the number of printed layers of negative and positive electrode respectively, V_N & V_P denote the volume of ink ejected in printing one layer, x_N & x_P denote the concentration of Zn and MnO_2 in the negative and positive electrode respectively, C_N & C_P denote the theoretical specific capacity of Zn and MnO_2 respectively. Here $V_N = V_P$, x_N = 8 mg/ml & x_P = 10 mg/ml, C_N = 820 mAh g^{-1} & C_P = 308 mAh g^{-1}. Putting all these values in Eq. (S4) gives a ratio of n_P/n_N ~ 2.

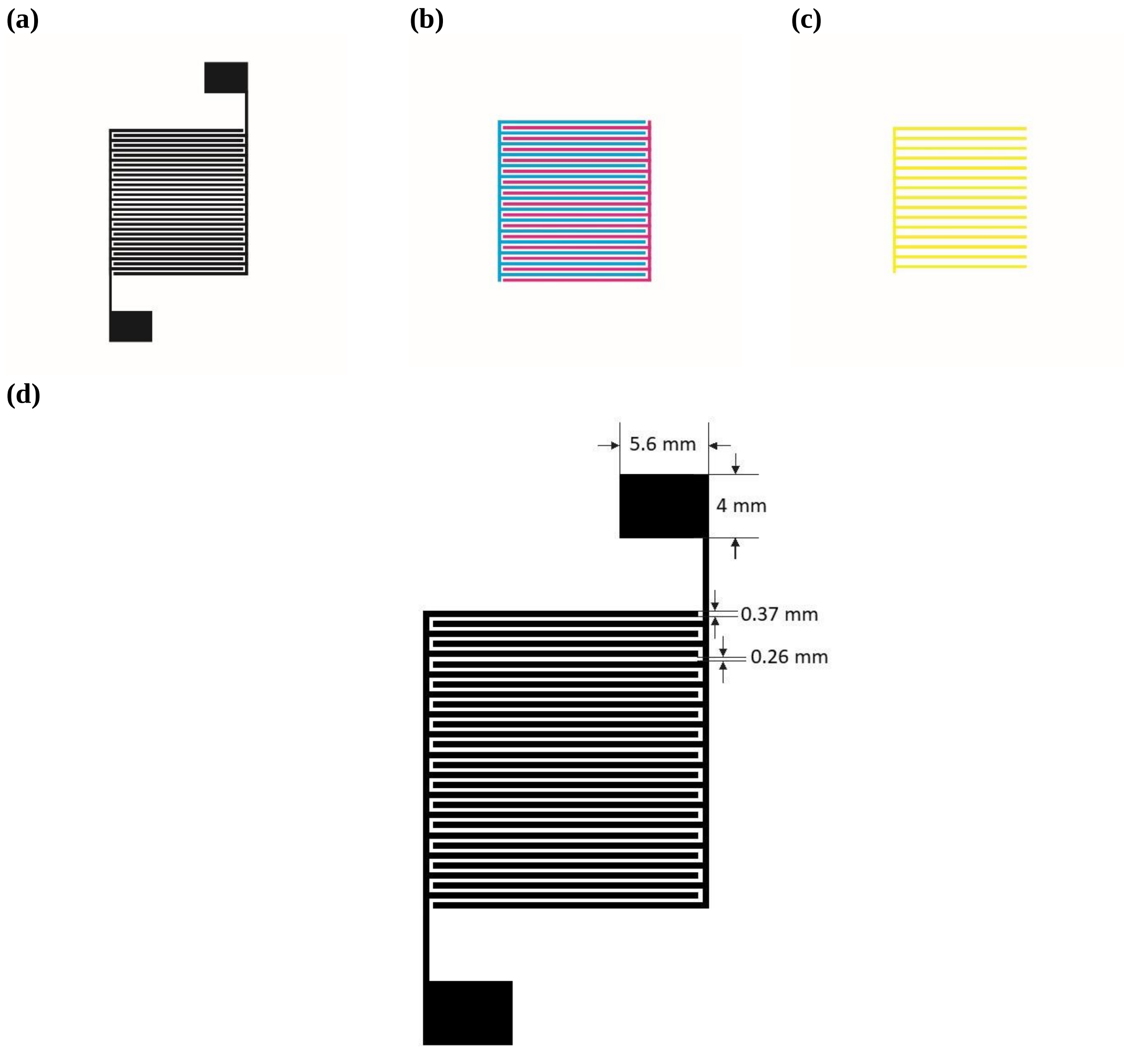

Fig. S1 Digital pattern and color sequence for printing (a) current collector (b) negative, positive electrode and (c) curing ink. (d) The various dimensions used for printing the IPZIB.

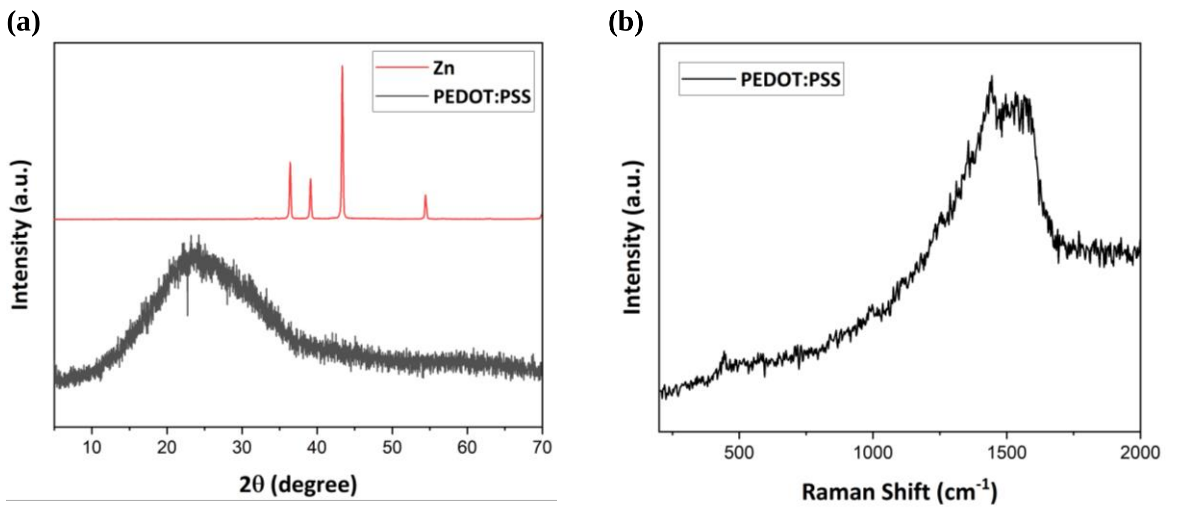

Fig. S2 (a)XRD pattern of Zn nanoparticles and PEDOT:PSS used in this work. (b) Raman spectrum of PEDOT:PSS used in this work.

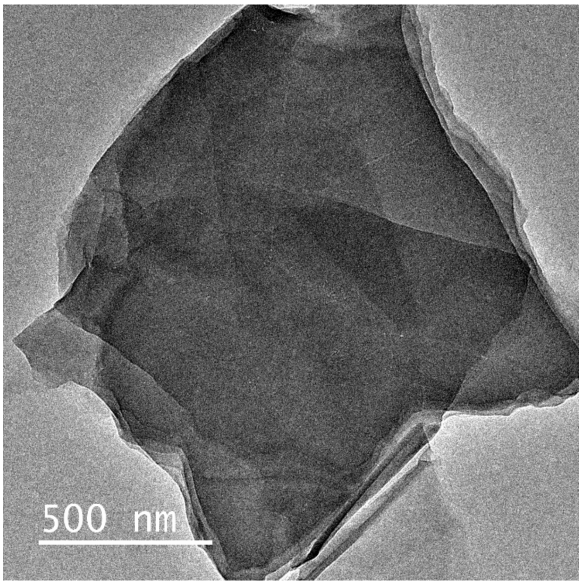

Fig. S3 TEM image of the synthesized rGO sheets.

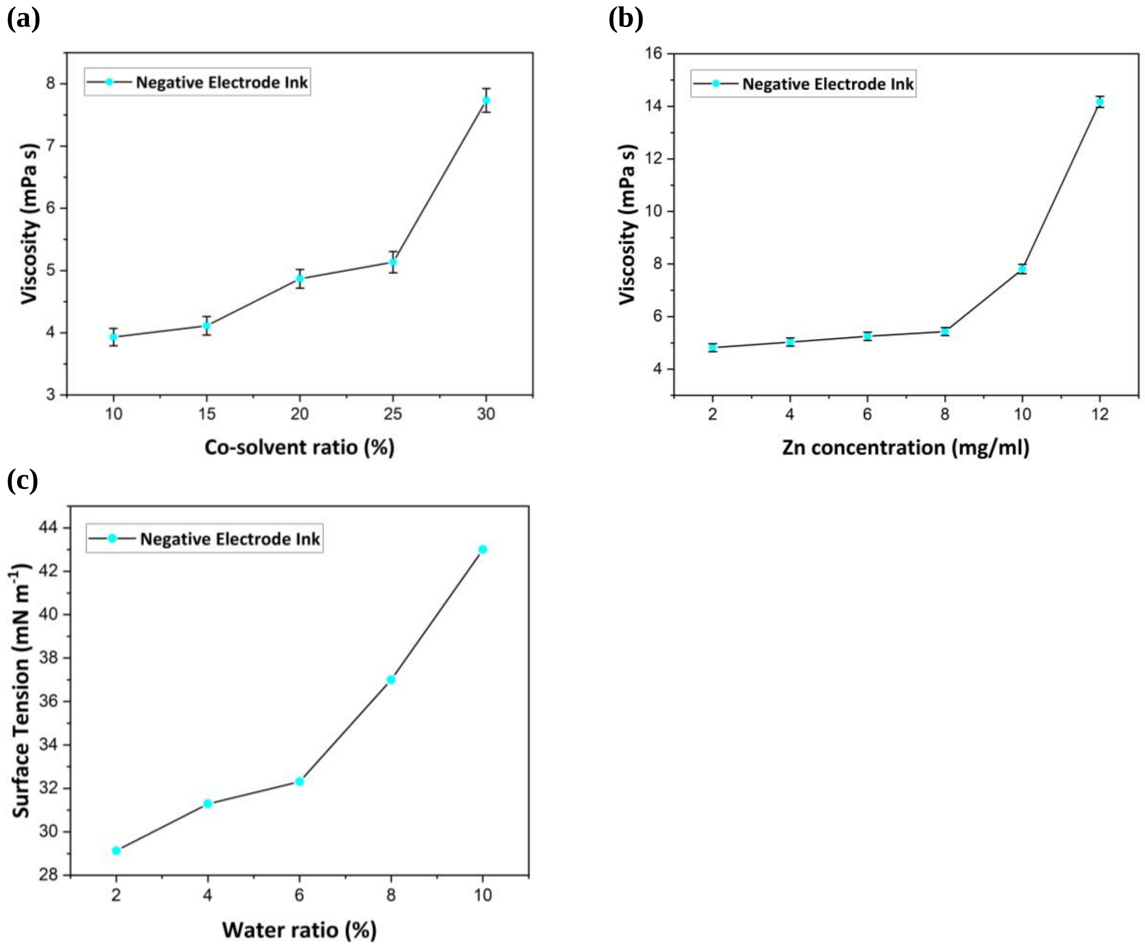

Fig. S4 Change in dynamic viscosity of the negative electrode ink with (a) volumetric percentage of the co-solvent and (b) Zn concentration. (c) Change in surface tension of the negative electrode ink with volumetric percentage of water.

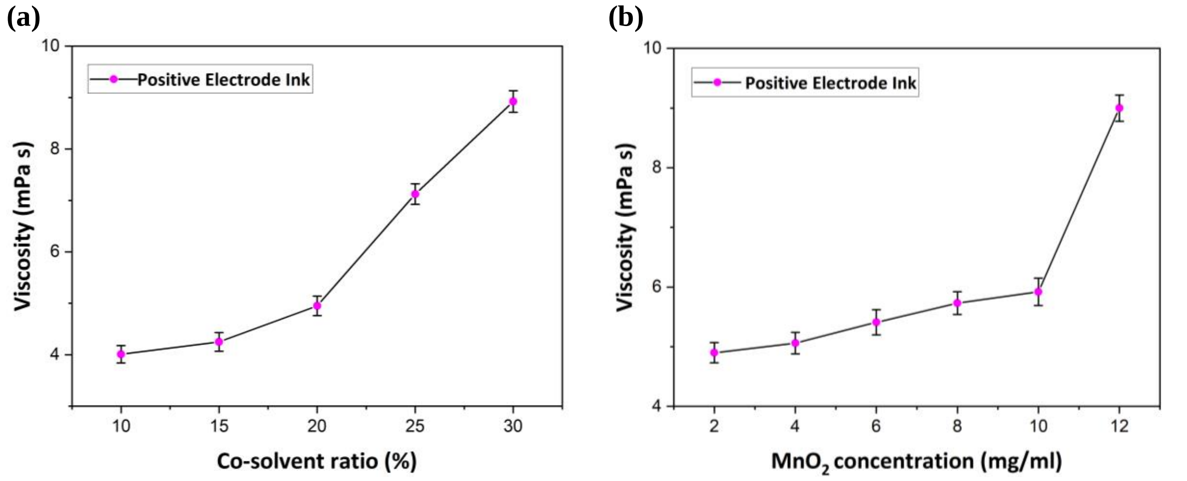

Fig. S5 Change in dynamic viscosity of the positive electrode ink with (a) volumetric percentage of the co-solvent and (b) MnO_2 concentration.

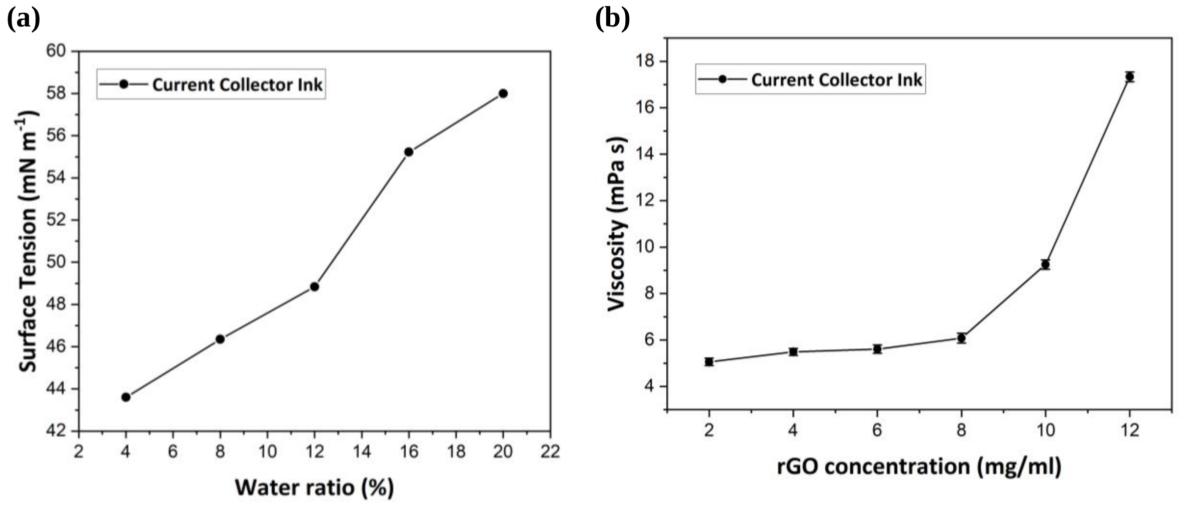

Fig. S6 (a) Change in surface tension of the current collector ink with volumetric percentage of water. (b) Change in dynamic viscosity of the current collector ink with rGO concentration.

**Table S1.** Density, viscosity, surface tension and the corresponding Fromm number for all the prepared inks.				
Ink	Density (kg/m^3)	Viscosity (mPa s)	Surface tension (mN m^{-1})	Fromm number (Z)
Negative electrode ink	948	5.43	43	5.26
Positive electrode ink	1087	5.92	57	5.95
Current collector ink	1063	6.08	58	5.78
Curing ink	1006	5.1	46	5.97

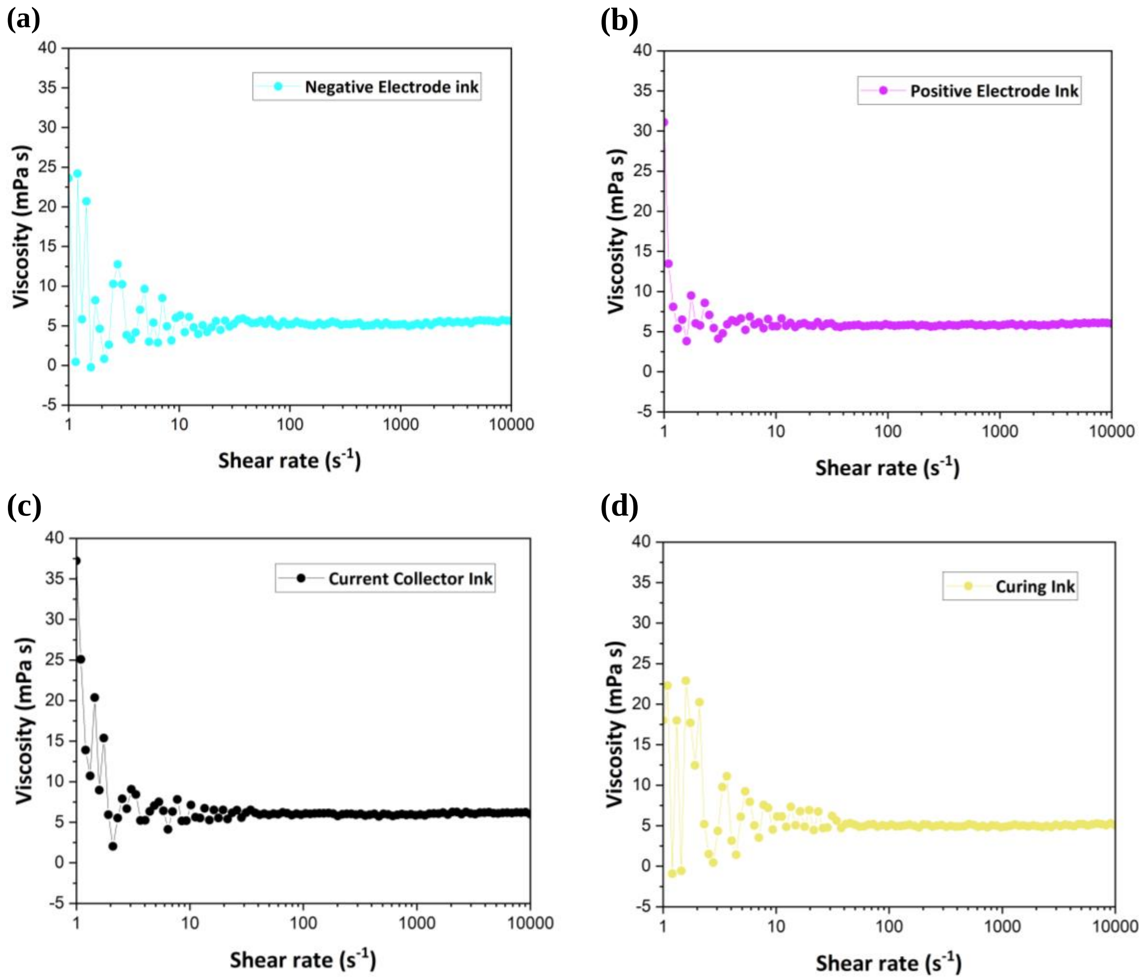

Fig. S7 Variation in the dynamic viscosity of the (a) Negative electrode (b) Positive electrode (c) Current collector and (d) Curing ink with shear rate.

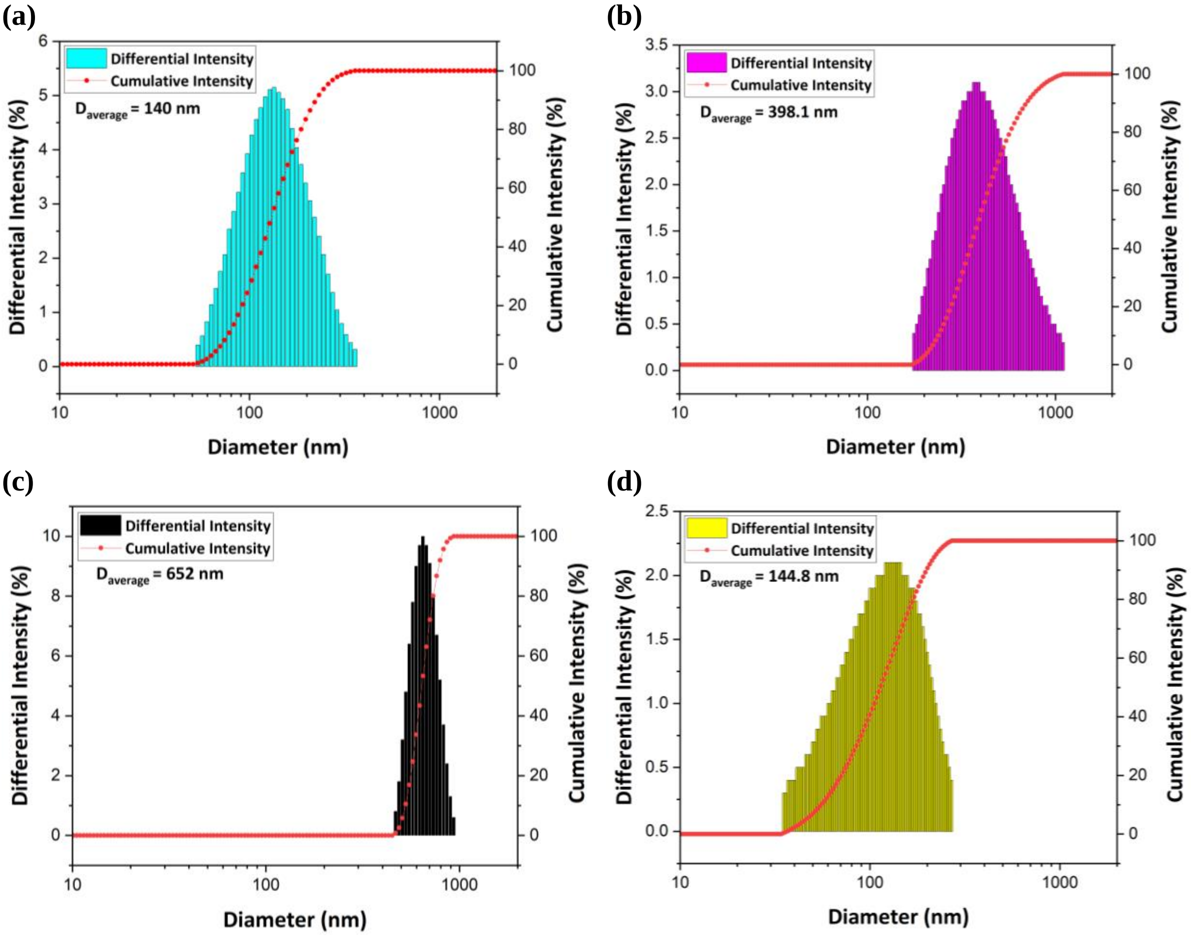

Fig. S8 Particle size distribution in the (a) Negative electrode (b) Positive electrode (c) Current collector and (d) Curing ink by DLS.

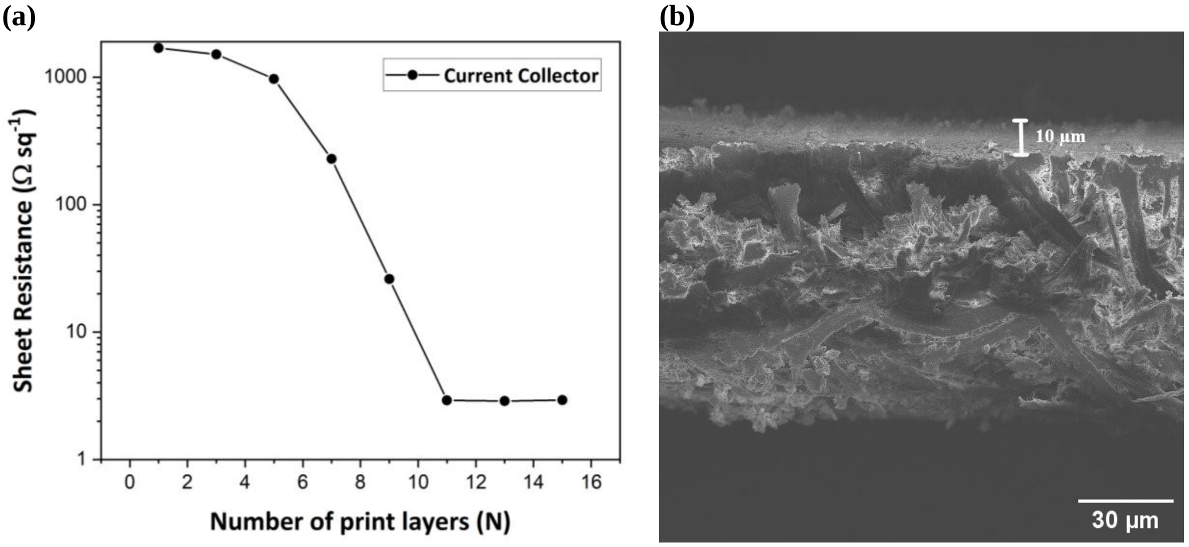

Fig. S9 (a) Change in sheet resistance of the printed current collector with the number of print layers. (b) SEM image of the current collector cross-sectional thickness with 11 printed layers.

Table S2. Change in specific discharge capacity of the IPZIB at a current density of 500 mA g^{-1} with different number of printed layers of the negative and positive electrode.

Negative electrode layers	Positive electrode layers	Specific discharge capacity (mAh g^{-1})
1	2	123.30 ± 1.7
2	4	199.70 ± 1.4
3	6	230.45 ± 1.1
4	8	221.07 ± 0.8
5	10	215.79 ± 0.6

Table S3. Change in sheet resistance of the printed Zn electrode with acetic acid concentration in the curing ink.

Acetic acid conc (wt%)	Sheet Resistance (Ω sq^{-1})
0	1258.37
5	71.23
8	4.78
12	9.01

(a) (b)

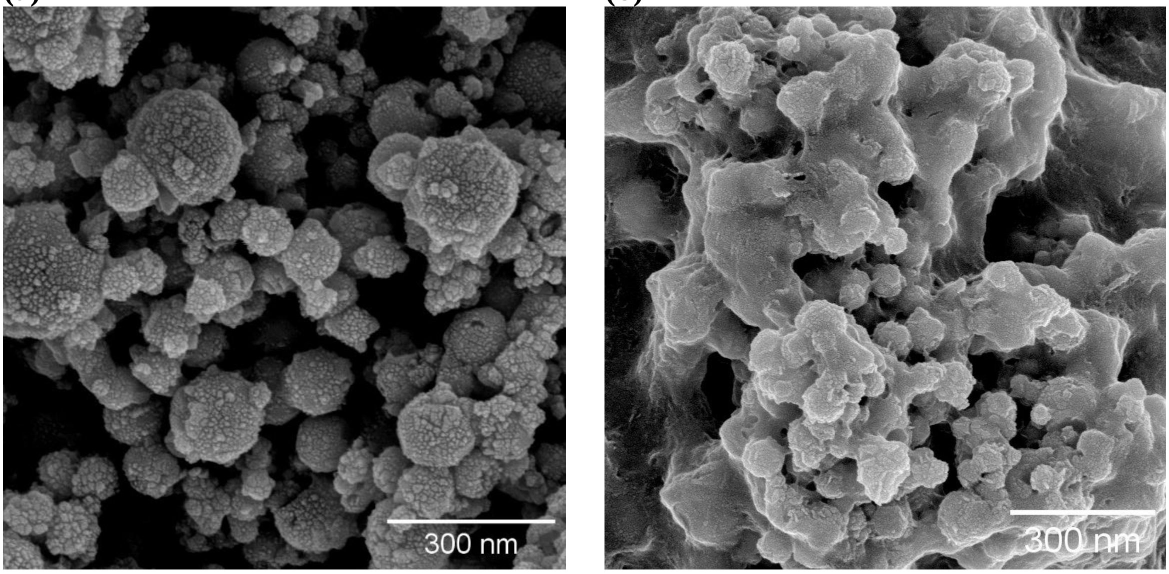

Fig. S10. SEM image of the (a) uncured and (b) the 8 wt% acetic acid cured Zn electrode.

(a)

Element	Atomic %
C K	28.19
O K	32.79
Zn L	39.02

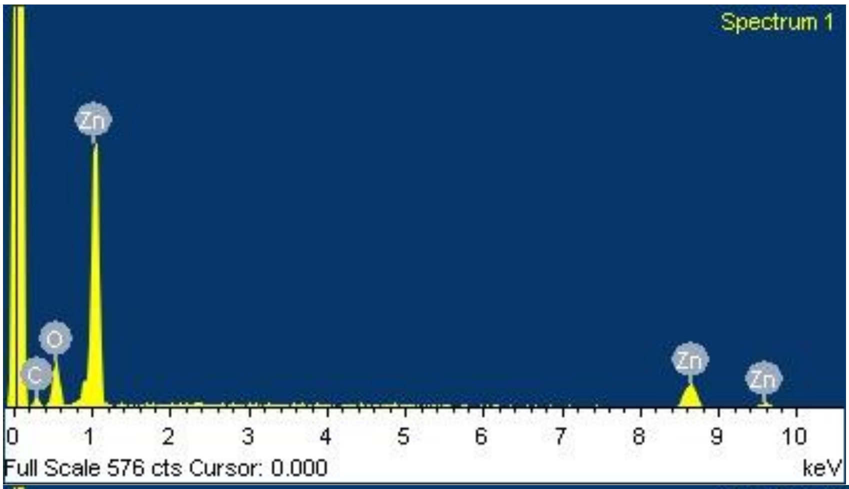

(b)

Element	Atomic %
C K	3.53
O K	6.77
Zn L	89.70

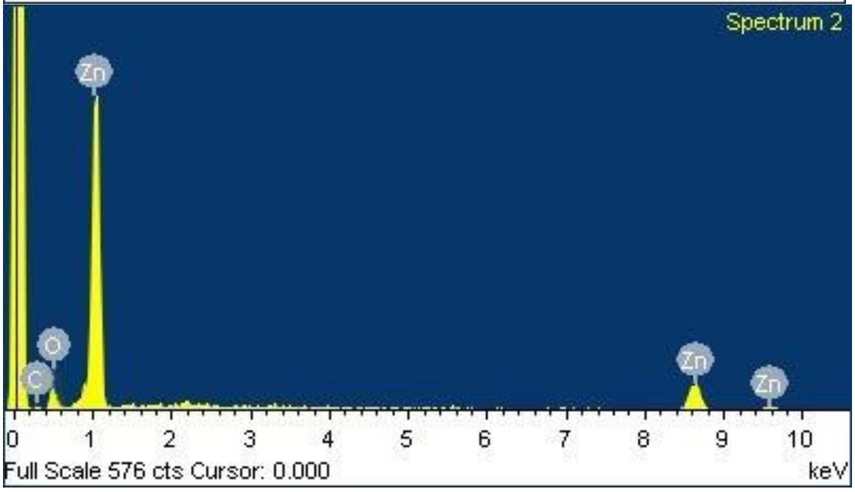

Fig. S11 EDX results of the (a) uncured and (b) cured Zn electrode.

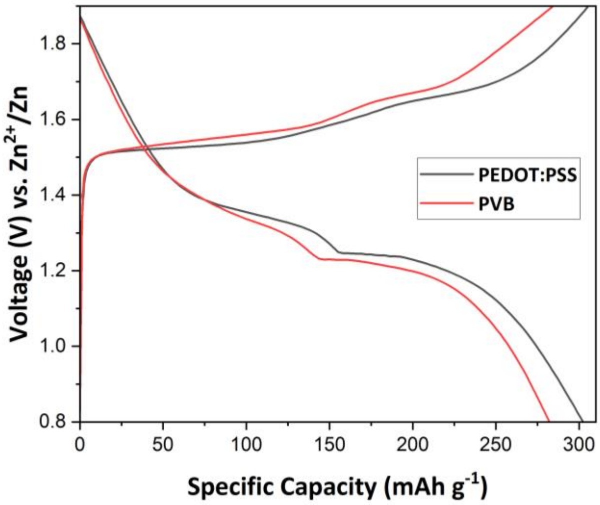

Fig. S12 Comparison of the Galvanostatic charge-discharge profile of the IPZIB with PEDOT:PSS and PVB as the binder.

Table S4. Specific discharge capacity and the corresponding capacity retention along with energy density and power density of the IPZIB at different current densities.

Current density (mA g^{-1})	Specific discharge capacity (mAh g^{-1})	Capacity retention (%)	Energy density (Wh kg^{-1})	Power density (W kg^{-1})
200	300.14	NA	330.154	220
300	281.01	93.63	309.111	330
400	262.11	87.33	288.321	440
500	233.04	77.64	256.344	550
600	207.21	69.04	227.931	660
700	186.53	62.15	205.183	770
800	168.31	56.08	185.141	880
900	143	47.64	157.300	990
1000	124.1	41.35	136.510	1100
1100	113.9	37.95	125.290	1210
1200	106.48	35.48	117.128	1320
1300	97.61	32.52	107.371	1430
1400	90.72	30.23	99.792	1540
1500	85.78	28.58	94.358	1650
200	281.7	93.86	309.87	220

Table S5. Change in R_{sol} and R_{ct} values of the fitted equivalent circuit after various cycles of charge-discharge.

Cycle number	R_{sol} (Ω)	R_{ct} (Ω)
1st	2.13	5.49
5th	2.16	6.17
20th	2.31	7.51
50th	2.61	9.89
100th	3.10	13.41

Table S6. The specific discharge capacity and the corresponding capacity retention at a current density of 600 mA g^{-1} of the IPZIB at various bending conditions.

Bending Condition	Specific discharge capacity	Capacity retention
Flat	206.11	NA
r = 10 mm	205.01	99.46
r = 5 mm	204.78	99.35
r = 0 mm	206.08	99.98
